# Hydrological and thermal responses of seeds from four co-occurring tree species from southwest Western Australia

**DOI:** 10.1093/conphys/coaa021

**Published:** 2020-04-30

**Authors:** Rajapakshe P V G S W Rajapakshe, Shane R Turner, Adam T Cross, Sean Tomlinson

**Affiliations:** 1 Centre for Mine Site Restoration, School of Molecular and Life Sciences, Curtin University, Perth, Western Australia 6845, Australia; 2 School of Molecular and Life Sciences, Curtin University, Perth, Western Australia 6845, Australia; 3 Kings Park Science, Department of Biodiversity, Conservation and Attractions, Kattidj Close, Kings Park, WA 6005, Australia; 4 School of Biological Sciences, Faculty of Science, The University of Western Australia, Crawley, WA 6009, Australia

**Keywords:** Conservation, drought stress, performance model, seed germination, thermal tolerance, threatened flora

## Abstract

Seed germination is a critical stage in the life cycle of most plants and is defined by specific tolerance thresholds beyond which rates and success of germination rapidly decline. Previous studies have demonstrated that widespread plant species commonly germinate over a broad range of temperatures and water stress levels, whereas range-restricted species often exhibit a narrower germination window in terms of temperature and moisture. We investigated the relationships of the key germination traits of maximum germination (*G*_max_) and time to 50% germination (*t*_50_) in response to temperature (5–35°C) and water stress (−1.5–0 MPa) in four co-occurring Western Australian native *Eucalyptus* species with widely varying biogeography. *Eucalyptus caesia* subsp. *caesia* and *E. ornata* exhibit a highly localized distribution and a narrow geographical range, being restricted either to granite outcrops or the upper slopes and tops of lateritic rises, respectively. These two species were compared with the two widespread and dominant congenerics *E. salmonophloia* and *E. salubris*. There was a distinctive hump-shaped response of *t*_50_ to temperature and an exponential response to water stress, characteristic of rate- and threshold-limited processes, but no consistent pattern in the response of *G*_max_. The four species were significantly different in their thermal performance of *t*_50_, with *E. caesia* and *E. ornata* displaying narrower thermal tolerance ranges than the two widespread species. In terms of mean final germination percentage, the two range-restricted endemic taxa exhibited higher lability in their response to thermal stress and drought stress compared to the two broadly distributed congenerics. These findings indicate a link between distributional extent, temperature and water stress tolerance and may have implications for identifying ecological filters of rarity and endemism.

## Teaser

Hydrological and thermal responses of seeds from four non-dormant co-occurring tree species from southwest Western Australia indicate a link between distributional extent, temperature and water stress tolerance and may have implications for identifying ecological filters of rarity and endemism.

## Introduction

High levels of biodiversity and endemism are often harboured in range-restricted niche habitats on rocky outcrops such as banded ironstone formations (BIFs) and granite outcrops (Porembski and Barthlott, 2000; Jacobi *et al.*, 2008; Gibson *et al.*, 2010). Opportunities for evolution in these habitats largely result from edaphic isolation from the surrounding vegetation matrices and the unique and highly localized environmental conditions commonly found in these niche landscapes (Porembski and Barthlott, 2000; Withers, 2000; Jacobi *et al*., 2008; Gibson *et al*., 2010). Consequently, the plant communities of rock outcrop habitats are often unique and comprise combinations of taxa that are regionally widely distributed as well as range-restricted ecological specialists that are highly adapted to various local microhabitats (Gibson *et al*., 2010; Porembski and Barthlott, 2012; Do Carmo and Jacobi, 2016). The result is that rock outcrop communities are generally speciose compared to adjacent vegetation on deeper soils (Main, 1997; Mares, 1997; Withers, 2000; Yates *et al.*, 2003; Schut *et al.*, 2014) and contribute significantly to regional biodiversity (Hopper and Gioia, 2004; Safford *et al.*, 2005; Jacobi and Fonseca do Carmo, 2008). For example, the granite outcrops of Western Australia host 17% of the flora native to the South Western Australian Floristic Region (SWAFR; Hopper and Gioia, 2004), including many range-restricted plant taxa that are threatened, yet granite outcrops occupy less than 1% of the land area in the SWAFR (Byrne and Hopper, 2008; Wege *et al.*, 2015).

Previous studies have highlighted topographic factors, edaphic isolation and climatic variables as major factors determining the distributional extent of narrow-range endemics (Yates *et al.*, 2004; Carta *et al.*, 2013; Tapper *et al.*, 2014a; Cross *et al.*, 2015a). For example, recent studies on the germination ecology of ephemeral taxa have revealed that hydrology regimes and hydroperiod are major ecological filters that determine species distributional range in temporary wetland habitats (Cross *et al*., 2015a; Cross *et al.*, 2015b; Carta *et al.*, 2013; Cross *et al.*, 2018). Widely distributed species in Mediterranean climatic regions commonly germinate over a relatively wide range of temperatures and water stress levels (Cochrane, 2017; Cochrane, 2018), whereas the germination response of range-restricted taxa has been shown in several species to be limited to a narrower window (Luna *et al.*, 2012; Turner *et al.*, 2018). Edaphic isolation and local topographic elements have been identified as driving forces of the patterns of plant diversity observed in rocky outcrop habitats such as BIFs and inselbergs (Jacobi *et al.*, 2007; Gibson *et al.*, 2010; Porembski and Barthlott, 2012; Do Carmo and Jacobi, 2016). Granite outcrops (and their immediate surroundings) represent a fine-scale mosaic of habitats, and where the ecophysiology of different elements of the floristic community might vary substantially (Withers, 2000; Byrne and Hopper, 2008; Tapper *et al.*, 2014b). Microhabitats in granite outcrop environments often harbour range-restricted and highly specialized species, as well as taxa that are widespread across different parts of the landscape (Hopper *et al.*, 1997; Withers, 2000). Exposed granite surfaces are characterized by high temperatures (particularly during summer) and low moisture availability due to high water runoff and limited capacity for moisture to soak into the subsurface environment (Withers, 2000; Porembski and Barthlott, 2012). However, following rainfall events, weathering of granite produces various highly localized, shaded, mesic microhabitats that retain water for periods of time including rock pools, crevices, gullies, talus and exfoliating sheets of granite where water collects and losses via evaporation and soil percolation are reduced (Wyatt, 1997; Withers, 2000; Liu *et al.*, 2007). The ecological filters underlying patterns of plant diversity in outcrop habitats are yet to be clearly identified and understood (Byrne and Hopper, 2008). However, the substantial proportion of range-restricted plant species endemic to rock outcrops suggests that the traits enabling these plant taxa to persist and flourish in their rocky niche may consequently reduce their competitiveness in other environments (Byrne and Hopper, 2008; Anacker *et al.*, 2011; Tapper *et al*., 2014b), and these warrant further investigation.

The transition from seed to seedling represents one of the most critical stages of the plant life cycle (Lloret *et al.*, 2004; James *et al.*, 2013). Seeds are therefore highly adapted to their habitat in order to maximize recruitment success, as essentially seeds have only one attempt at successfully transitioning from a seed to a viable and healthy seedling (Walck *et al.*, 1997; Tweddle *et al.*, 2003; Luna *et al.*, 2012). Consequently, the environmental requirements for dormancy alleviation and seed germination are usually definable, highly nuanced and species-specific (Turner *et al.*, 2018). Seed germination occurs in response to specific combinations of environmental cues above critical thresholds with two of the most important being temperature and soil moisture (Bell, 1994; Bell *et al.*, 1995; Merritt *et al.*, 2007). It is reasonable to expect that range-restricted species, and particularly species occurring only in specific microhabitats such as rocky outcrops, may have narrow germination niches as these habitats provide environments that are likely to differ markedly from other parts of the landscape (Turner *et al.*, 2018; Elliott *et al.*, 2019). Consequently, investigation of the germination ecology of seeds from range-restricted and ecologically specialized flora should be a principle area of research to better understand their demographic limitations which may assist with their ongoing conservation and management (Luna *et al.*, 2012; Clemente *et al.*, 2017). Furthermore, identifying some unifying theoretical constraints to seed germination is essential for constructing *a priori*, mechanistic hypotheses underpinning these demographic limitations.

**Table 1:** Seed traits of selected *Eucalyptus* species used in this study. *E. caesia* subsp. *caesia* is the most tightly distributed species, occurring in only three IBRA regions^*^, followed by *E. ornata* (2 IBRA regions), *E. salmonophloia* (eight IBRA regions) and *E. salubris* (nine IBRA regions)

**Species**	**Collection location**	**Collection date**	**1000-seed weight (mg)**	**X-ray fill (%; *n* = 100)**	**Viability (%; *n* = 20) (tetrazolium test)**	**Germinability (%) of viable seeds** ^**^ **(*n* = 25)**
***Eucalyptus caesia subsp. caesia***	Kuender, WA	01/2018	2552	96	90	93
***Eucalyptus ornata***	Kondinin, WA	11/2008	1843	100	100	100
***Eucalyptus salmonophloia***	Kondinin, WA	09/2017	209	93	100	100
***Eucalyptus salubris***	Kondinin, WA	03/2016	574	100	90	100

^*^Interim Biogeographic Regionalisation for Australia (Thackway and Cresswell, 1997) ^**^Initial germination success of filled seeds was assessed by incubating 25 seeds of each species on moist germination paper in Petri dishes under constant darkness at 10, 15, 20, 25, 30 and 35°C followed by daily scoring of germination rate.

There have been efforts made to develop models of seed germination in relation to temperature and water stress (Bradford, 2002), but these have been heavily data-referential, and have not been consistent with the theoretical underpinnings of the wider thermal performance literature (Angilletta Jr *et al.*, 2009). As such, the statistical fitting is potentially over-simplified, and the resulting parameters may be inaccurate and difficult to place in a broad theoretical context. According to the collision theory of chemical kinetics, reaction rates increase exponentially with increasing temperature (Gates, 2016). However, metabolic reactions are catalyzed by enzymes that have a specific thermal threshold beyond which they denature (Peterson *et al.*, 2007). The interaction of these two processes implies a rapid increase in physiological performance up to a critical threshold, beyond which performance rapidly declines as chemical reactions cease to be catalyzed by the denaturing enzymes. Therefore thermal performance curves of enzymes are hump shaped and distinctly asymmetrical (Angilletta Jr, 2006; Tomlinson, 2019), which is an important trait conspicuously absent in published early models (e.g. Bradford, 2002). There is also variability in the breadth of these responses that has evolutionary and ecological value (Huey *et al.*, 1989). Seeds of widely distributed flora are expected to have broad thermal tolerance ranges (eurythermy) to match the breadth of climatic conditions across their distributions, while range-restricted congenerics are expected to be thermally specialized (stenothermy; Debat and David, 2001; Ghalambor *et al.*, 2007). In this manner, seed germination is consistent with general models of stenothermy and eurythermy (Seebacher and Franklin, 2005). However, there is a shortage of studies that have incorporated these well-established principles of chemical kinetics to quantify the impact of thermal stress on germination response in the literature. Further, there is a major shortage of research data on how a species distributional range affects germination response to water stress. Given that rocky outcrop habitats comprise highly variable microclimates and that these landscapes can be very hot and dry for much of the year, especially in the lower rainfall regions of Western Australia, the optimal performance windows might reflect highly specific local adaptations and thus provide some insight concerning *in situ* recruitment processes (Byrne and Hopper, 2008; Tapper *et al*., 2014b). This study aimed to compare the germination responses of two range-restricted granite outcrop specialist species with those of two widely distributed co-occurring taxa to address the following two research questions: (i) Are seeds of non-dormant range-restricted species more sensitive to incubation temperature compared to common congeneric taxa? And (ii) Are seeds of non-dormant range-restricted species more sensitive to water stress compared to common congeneric taxa?

## Materials and methods

### Species selection and sourcing

We conducted this study using four readily germinable, non-dormant species of *Eucalyptus* endemic to southwest Western Australia. Species were selected to eliminate the potential confounding effect of seed dormancy on examining seed germination responses. We selected seeds of two range-restricted species native to either granite outcrop habitats (*E. caesia* Benth. subsp. *caesia*) or laterite ridges (*E. ornata* Crisp) and two widely distributed congeneric species (*E. salmonophloia* F.Muell. and *E. salubris* F.Muell.). Seeds were either freshly harvested from wild populations (*E. caesia*) or obtained from a commercial seed supplier (*E. ornata*, *E. salmonophloia* and *E. salubris—*Nindethana Seed Company, King River, Western Australia) with known collection locations and dates of collection (Table 1). *Eucalyptus caesia* Benth. subsp. *caesia* and *E. ornata* are range-restricted mallees that are gazetted as priority 3 and 4 respectively (W.A. Herbarium, 2018) so are of some conservation concern (Coates *et al.*, 2001). *Eucalyptus. caesia* subsp. *ceasia* is distributed across 25 populations in the Avon Wheatbelt, Coolgardie and Mallee (Bezemer *et al.*, 2019), whereas the range of *E. ornata* is limited to five populations in the Avon Wheatbelt and the Mallee IBRA (Interim Biogeographic Regionalisation for Australia) Regions (Thackway and Cresswell, 1997). In contrast, *E. salmonophloia* and *E. salubris* are common, widely distributed dominant mallees native to south west Western Australia (Yates *et al.*, 1994). Their habitats are diverse and include undulating low hills, plains and slopes surrounding granite outcrops. The distributional range of *E. salmonophloia* and *E. salubris* extends from the relatively mesic Mediterranean SWAFR (South West Australia Floristic Region) to the semi-arid IBRA regions such as Coolgardie (Yates *et al.*, 1994). Seed accessions used in this experiment were collected in 2008 (*E. ornata*), 2016 (*E. salubris*), 2017 (*E. salmonophloia*) and 2018 (*E. caesia*) from locations within ~ 50 km of each other (Table. 1). Seeds from all species were stored under cool, dry conditions until utilized in this study.

### Seed quality

Prior to experimentation, seeds were stored in a controlled environment (15°C and 15% relative humidity) at the Biodiversity Conservation Centre, Kings Park, Western Australia. We used a vacuum aspirator (SELECTA BV Gravity Seed Separator, the Netherlands) to separate seeds from chaff. For each test species percentage seed fill was determined by X-ray analysis of 100 seeds (MX-20 digital X-Ray cabinet, Faxitron, USA). A seed containing a fully developed embryo and endosperm can be identified by uniform white/grey coloration (filled tissue), whereas the absence of these tissues indicates a lack of seed fill (Erickson *et al.*, 2016).

For seeds that were filled, seed viability was also investigated using Tetrazolium staining (Lakon, 1949). Reduction of 2,3,5-triphenyltetrazolium chloride (C_19_H_15_N_4_Cl) by dehydrogenase enzymes present in live tissues produces an intense pink colour, indicating that a seed is metabolically active, and thus viable (Lakon, 1949; Jeremiah *et al.*, 2002). Samples of 20 seeds per species were horizontally dissected and exposed to 1% tetrazolium for a period of 4 h at 25°C. We used stained seeds to calculate percentage viability of seed lots (Table 1).

### Temperature tolerance

**Figure 1:**
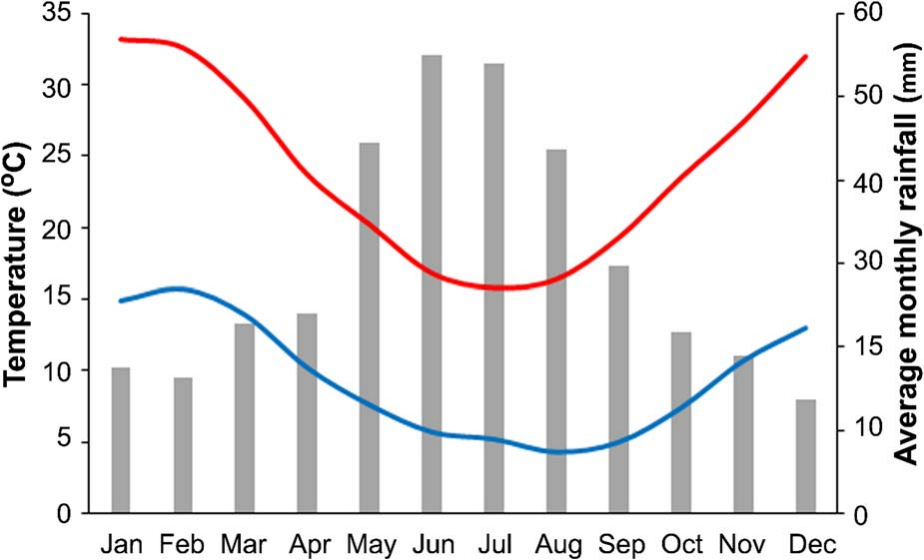
Long-term climatic data for the Kondinin meteorological station, encompassing average monthly maximum (red line) and minimum (blue line) temperatures and average monthly rainfall (grey bars).The temperature range of the experimental trials conducted here span the full temperature range in the region, from 5 to 35°C

To assess the germination response of seeds to temperature, we placed eight replicates of 25 seeds for each species on moist (9 ml of distilled water per petri dish) 84 mm germination paper (Advantec, Dublin, CA, USA) in 90-mm plastic Petri dishes and incubated at 5, 10, 15, 20, 25, 30 and 35°C (1400 total seeds per species). These conditions encompass a broad range of the temperatures reported for the location of the test species for all seasons (Bureau of Meteorology, 2018; Fig. 1). Seeds were surface sterilized with 2% (w/v) calcium hypochlorite (Ca[OCl]_2_) under vacuum (−70 kPa) for 30 min and washed with sterile deionized water three times for several minutes per wash prior to plating. We conducted seed plating under sterile conditions in a laminar flow cabinet. Petri dishes were sealed with plastic wrap to prevent moisture loss during the incubation period. Petri dishes were also covered with aluminium foil to eliminate the potential confounding effect of light on germination (Bell, 1994; Ruiz-Talonia *et al.*, 2018). The temperature inside the incubators was recorded once an hour using iButton data loggers (Maxim Integrated™, San Jose, USA) placed in the middle of each stack of eight petri dishes (see Supplementary Material). We scored germination as radicle emergence greater than 2 mm, and plates were scored four days a week for a period of 28 days.

### Water stress tolerance

To test the effect of water stress on germination, we placed seeds in 90-mm plastic Petri dishes on seed germination papers as previously described infused with different concentrations of polyethylene glycol 8000 (PEG) solution (9 ml of PEG per petri dish) following Michel (1983). Plates were incubated at a constant favourable incubation temperature (20°C), determined from temperature tolerance experiments. We exposed eight replicates of 25 seeds for each species to water stress levels of 0, −0.10, −0.20, −0.40, −0.70, −1.00 and −1.50 MPa (1400 total seeds per species). Seeds were surface sterilized as previously described prior to plating, and Petri dishes were tightly sealed with plastic wrap and incubated in constant darkness with iButtons (Maxim Integrated™, San Jose, CA, USA) placed on the middle of each Petri dish stack to measure incubation temperature as previously described. We scored germination as radicle emergence greater than 2 mm, and plates were scored four days a week for a period of 28 days.

## Statistical analysis

### Germination modelling

Traditional attempts to identify critical thresholds of seed germination utilize binominal logistic regression to linearize the relationship between treatments and germination response (Ashford *et al.*, 1970; Bradford, 2002). We adapted a non-linear regression approach (Ritz and Streibig, 2008) that is not yet common in studies of seed biology to assess the effect of incubation temperature and water stress on germination response. The main advantage of the non-linear curve-fitting approach we have used is that it does not compress the natural variance structure of the data in the way that linearization does and only fits the number of parameters that define the model. Therefore, since the risk of overfitting to the data is substantially reduced, non-linear regression is more objective and parsimonious than generalized additive modelling (GAM) approaches (Tomlinson, 2019). First, we assessed the relationship describing the germination response over time for each experimental temperature using curvilinear log-logistic germination models (Lewandrowski *et al.*, 2017; Tarszisz *et al.*, 2017). The *drc* package (Ritz *et al.*, 2012) was used to fit a three-parameter log-logistic function to germination data in the *R* statistical environment (R Core Team, 2013);

(1) \begin{equation*}\mathrm{germination}=\frac{G_{\mathrm{max}}}{1+{\frac{\mathrm{time}}{t_{50}}}^b}\end{equation*}

where *G*_max_ is the upper limit for the germination rate, and the lower limit of germination rate is assumed to be 0 (Lewandrowski *et al*., 2017). The function also calculates a point around which the equation is symmetrical, *t*_50_, which is an estimate of the time required for 50% of the seeds (as a percentage of *G*_max_) to germinate and *b* indicates the slope of the germination function at *t*_50_. First, we resolved a convergent common curve for the number of germinants over the number of seeds incubated for all of species under all temperature regimes. By grouping this function by species and incubation temperature, unique values were fitted to the parameters of the function to produce several permutations of the basic model. We utilized the *AICcmodavg* package (Mazerolle, 2013) to assess the explanatory power of ‘species’ and ‘incubation temperature’ as factors contributing to variability in germination response (in terms of *t*_50_ and *G*_max_) by comparing each permutation with the common curve using the Akaike information criterion (Burnham *et al.*, 2002). The log-logistic model grouped into unique species and temperature categories was utilized to estimate *t*_50_ and *G*_max_ values for each replicate of all species incubated under different treatment regimes. We used model estimates for *b*, *G*_max_ and *t*_50_ to calculate time (in days) to reach *G*_max_ for all replicates exposed to different treatment regimes.

### Temperature tolerance

The precision of curvilinear modelling is dependent upon assumptions related to the shape of the curve (Tomlinson, 2019). Although thermal performance generally shows an asymmetrical increase with a single peak (Angilletta Jr, 2006; Peterson *et al*., 2007), appropriate non-linear thermal performance functions are yet to be described for seeds (Yan and Hunt, 1999). Therefore, we estimated unimodal asymmetrical model fits for the 1/*t*_50_ estimates for our thermal response data using a thermal performance function which has been described by Yan and Hunt (1999) for the temperature response of maximum rate of growth in plants;

(2) \begin{align*}{r}_{\mathrm{max}}={R}_{\mathrm{max}}\ \left(\frac{T_{\mathrm{max}}-T}{T_{\max -}{T}_{\mathrm{opt}}}\right){\left(\frac{T}{T_{\mathrm{opt}}}\right)}^{\frac{T_{\mathrm{opt}}}{T_{\mathrm{max}}-{T}_{\mathrm{opt}}}}\end{align*}

where *r*_max_ is the maximum germination rate at any temperature (*T*), *T*_opt_ is the optimum temperature for germination at the peak of the performance function, *T*_max_ is the limit of thermal tolerance, where germination ceases, and *R*_max_ is the asymptotic maximum germination rate at *T*_opt_. Henceforth, 1/*t*_50_ will be referred to as the thermal performance of maximum germination rate, *r*_max_, as a proxy for the speed of germination across a temperature gradient. The thermal performance of maximum germination rate at the optimum temperature is characterized as *R*_max_. A major advantage in this approach is that each parameter of the above equation can be directly translated into a factor that has biological meaning. Therefore, these parameters can be readily compared across taxa to gain insights into patterns of variability in germination response.

### Water stress tolerance

In the same way that seed germination should be inhibited by the thermal performance of enzyme function at specific thermal thresholds, it should be impeded by reduced water availability as well, and *t*_50_ for seeds should escalate exponentially with increasing water stress up to a species specific threshold at which the low water potential of the external environment prevents imbibition (Bradford, 2002). A pattern of exponential increase in *t*_50_ in response to increasing water stress is consistent with previous studies on multiple taxa native to the SWAFR (Cochrane, 2018). Consequently, we selected an exponential function with the minimal number of parameters required to simulate the water stress response of non-dormant seeds to fit the *t*_50_ estimates for our water stress response data;

(3) \begin{equation*}{t}_{50}={g}_0+{e}^{\left\{k\times \left(w+{w}_c\right)\right\}}\end{equation*}

where *t*_50_ is the time required to reach 50% germination under any water stress level, *g*_0_ is the base value of *t*_50_ prior to the beginning of its exponential increase, *k* is a scaling exponent and *w_c_* is the critical water stress level at which *t*_50_ begins to escalate exponentially.

### Unique parameterization

We fitted the appropriate physiological functions (thermal performance or hydrological performance) to the log-logistic model estimates using the *thermPerf* package (Bruneaux, 2017) in the *R* statistical environment (R Core Team, 2013) to identify a global model. Subsequent to this we employed the *nls* function to fit unique values to the parameters of the performance function on the basis of species, following Ritz and Streibig, (2008) to parameterize unique values of *R*_max_, *T*_opt_, *T*_max_, *g*_0_ and *w_c_* for each species in terms of *t*_50_ and *G*_max_.

**Figure 2:**
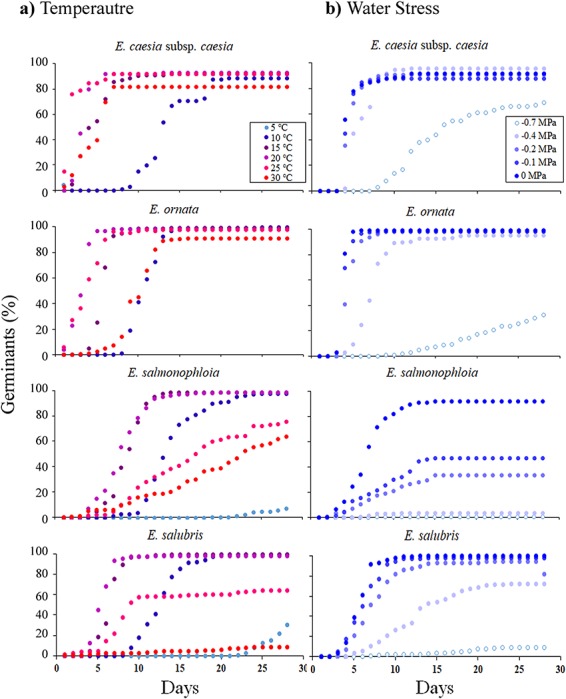
Mean cumulative germination percentage for replicates of seeds of four Western Australian *Eucalyptus* species following exposure to an increasing range of temperatures (**a**) and water stress levels (**b**) incubated in constant darkness for 28 days. Eight replicates of 25 seeds were used for each treatment. Error bars depicting standard error of the mean have been omitted for clarity

## Results

### Germination modelling

**Figure 3:**
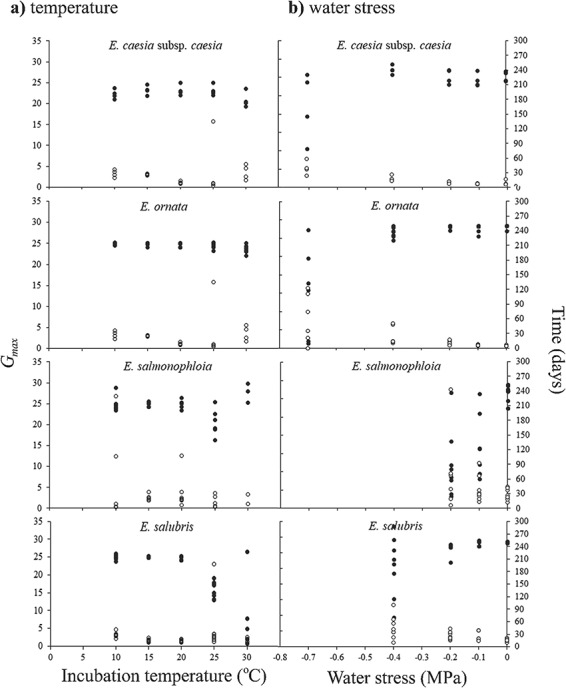
Estimates for maximum germination (*G*_max_) and time to reach *G*_max_ predicted by a three-parameter log-logistic function for the germination responses of four Western Australian *Eucalyptus* species following incubation in constant darkness at varying temperature regimes (**a**) and water stress levels (**b**). Black dots depict *G*_max_ estimates and non-shaded dots represent time to reach *G*_max_ for each replicate of seeds following exposure to different treatment regimens. Eight replicates of 25 seeds were used for each treatment

The two range-restricted species displayed higher final germination percentages over a wider range of temperatures than the two broadly distributed taxa (Fig. 2a). Both *Eucalyptus caesia* subsp*. caesia* and *E. ornata* exhibited relatively constant high final germination percentages (>80%) from 10 to 30°C, while final germination percentages of *E. salmonophloia* and *E. salubris* decreased from 98% to < 76% at 25°C (Fig. 2a). For the two range-restricted taxa, the minimum final germination percentage was observed at 10°C, whereas for the two widely distributed taxa minimum final germination occurred at 5°C (Fig. 2a). For all taxa except *E. caesia* subsp. *caesia*, the maximum temperature at which germination occurred was 35°C (Fig. 2a). Within the range of 15–25°C, estimated time to reach *G*_max_ was ≤ 30 days for most replicates of the four species (Fig. 3a). For *E. caesia* subsp. *caesia* and *E. ornata*, deviation from favourable temperatures increased variability in *G*_max_ and lengthened the time required to reach *G*_max_ (Fig. 3a). However, for *E. salubris* the time to reach *G*_max_ was relatively consistent across 10–30°C (Fig. 3a).

The range-restricted *E. caesia* subsp. *caesia* and *E. ornata* were more tolerant of water stress than the two widely distributed taxa, in terms of final germination percentage. The final germination percentage of the two range restricted taxa exceeded 90% even at −0.4 MPa (Fig. 2b). Conversely, the final germination of *E. salmonophloia* and *E. salubris* seeds decreased to < 80% at −0.1 and −0.4 MPa, respectively (Fig. 2b). For *E. caesia* subsp. *caesia* and *E. salmonophloia*, the highest stress level at which germination occurred was −1 MPa, whereas for *E. ornata* and *E. salubris* germination was not observed below −0.7 MPa (Fig. 2b). For all tested species, estimates for time to reach *G*_max_ and variability of these estimates increased with rising water stress (Fig. 3b).

### Temperature tolerance

**Figure 4:**
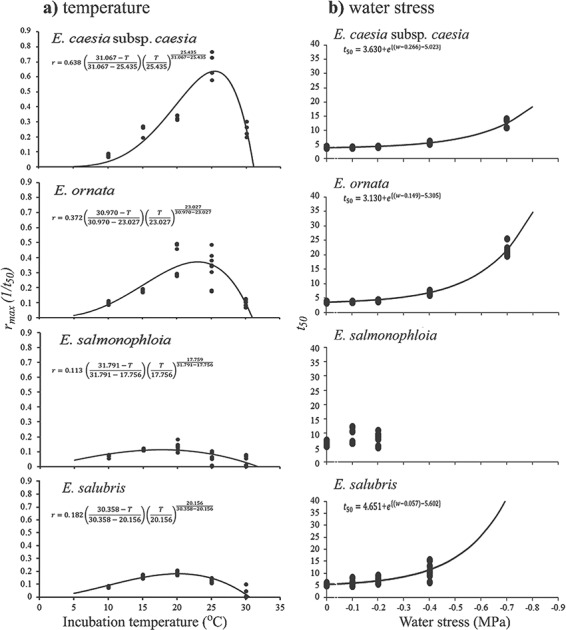
Thermal performance and water stress tolerance in four Western Australian *Eucalyptus* species in terms of time to reach 50% germination (*t*_50_). (**a**) Dots represent 1/*t*_50_ estimates for each replicate of seeds after exposure to different temperature regimes and the smooth lines represent the permutations of the thermal performance curve fitted to the 1/*t*_50_ estimates (*r*_max_) of each species. Coefficients for the permutations of the most parsimonious thermal performance function resolved on the basis of species are displayed. (**b**) Dots represent *t*_50_ estimates for each replicate of seeds after exposure to different water stress regimes and the smooth lines represent the exponential models fitted to the *t*_50_ estimates of each species. Coefficients for the most parsimonious water stress tolerance model fitted to the germination response data of each species are displayed. Eight replicates of 25 seeds were used for each treatment

The log-logistic curve incorporating both species and temperature regime was the best model to fit our thermal response data (AICc = 25106.87, *df* = 76, residual deviance = 2.608; Supplementary Material) indicating that both ‘species’ and ‘incubation temperature’ were factors that contributed to variability in germination response (Supplementary Material). The log-logistic curve could not be fitted to the germination response data for 5 and 35°C since final germination percentages were very low (<31%) at these temperatures (Fig. 2a). The distribution of the *r*_max_ values estimated by the log-logistic model for each species-by-temperature grouping across 10–30°C was hump shaped, increased exponentially with increasing temperature up to a peak, beyond which it decreased rapidly (Fig. 4a). The most parsimonious model resolved unique *R*_max_, *T*_opt_ and *T*_max_ values defining the thermal performance of *r*_max_ for each species (Equation 2, Fig. 4a). For *E. caesia* subsp. *caesia* and *E. ornata*, estimated *T*_opt_ values were 25.4 ± 0.25 and 23.0 ± 0.37°C respectively, whereas for *E. salmonophloia* and *E. salubris* estimates for *T*_opt_ were 17.7 ± 1.94 and 20.1 ± 0.97°C, respectively (Fig. 4a). The two widely distributed species had broader thermal tolerance ranges than the two range-restricted taxa, apparently reflecting a higher level of physiological plasticity (Fig. 4a). For all tested species, estimated *T*_max_ was within the range of 30.5–32°C. A thermal performance function could not be resolved for the *G*_max_ estimates of the log-logistic model (Fig. 3a) because they were highly conserved across all experimental temperatures.

### Water stress tolerance

The best log-logistic function to fit our water stress response data was the permutation incorporating both species and water stress regime (the lowest AICc value = 18869.09, *df* = 61, residual deviance = 6.503; Supplementary Material). For *E. caesia* subsp. *caesia*, *E. ornata* and *E. salubris*, the *t*_50_ values estimated by the log-logistic model were relatively constant up to a threshold water stress level, which was followed by an exponential rise in *t*_50_ with increasing water stress (Fig. 4b). The exponential function fitted to the *t*_50_ estimates resolved *g*_0_, *k* and *w_c_* estimates for the global model. However, the exponential model failed to resolve water stress response profiles on the basis of species. Therefore, unique values were fitted to the function parameters for each species-by-water stress regime separately (Fig. 4b). For *E. caesia* subsp. *caesia*, *E. ornata* and *E. salubris*, estimated *w_c_* values were −0.266 ± 0.098, −0.149 ± 0.049 and − 0.057 ± 0.250 MPa, respectively (Fig. 4b). However, this exponential model could not be fitted to the *t*_50_ estimates for *E. salmonophloia* since final germination percentage declined to < 10% for water stress regimes lower than −0.2 MPa (Figs. 2b and 4b). Furthermore, the exponential function could not be fitted to the *G*_max_ estimates of the log-logistic model (Fig. 3b).

## Discussion

The results of this study demonstrate that the thermal performance of the four selected taxa in terms of *t*_50_ is hump-shaped, in accordance with established principles of thermal biology that germination response to temperature should resemble thermal performance curves of enzymes. The key elements captured by applying the Yan and Hunt (1999) model are the asymmetrical nature of the curves, and the ability to directly compare differences in the shape of these functions between different taxa. For example, our observations conform to the general models of stenothermy and eurythermy in that the two range-restricted endemic taxa exhibited narrower thermal tolerance ranges than their co-occurring congenerics in terms of *t*_50_. However, in terms of final germination percentage, the narrow-range endemics were more tolerant of thermal stress than the two widely distributed taxa. Our second hypothesis, that the range-restricted endemic taxa would be more sensitive to water stress, was not supported in terms of final germination percentage. However, it is not clear to what extent the four species differ in water stress tolerance in terms of *t*_50_.

### Temperature and water stress tolerance

The high seed viability that we observed is consistent with previous reports of high germination success in *Eucalyptus* species (a non dormant group; Baskin and Baskin, 2003) from across Australia when incubated under favourable thermal conditions (Bell *et al*., 1995; Ruiz-Talonia *et al*., 2018). According to the seed dormancy classification system proposed by Baskin and Baskin (2003), non-dormant species usually germinate within a period of 30 days under favourable environmental conditions. However, seed germination is a physiological process that is limited to a temperature range suitable for normal metabolic activity (Bell *et al*., 1995, Jiménez-Alfaro *et al.*, 2016). Bell *et al*. (1995) reported that in six species of *Eucalyptus* native to Western Australia, final germination percentage was highly variable, and Cochrane (2017) has reported that many *Eucalyptus* species native to southwestern Australia exhibit high plasticity to thermal stress in terms of final germination percentage. Our data did not provide strong support for these statements, in that, while temperature regimes beyond 10–20°C reduced mean final germination percentage in all species tested (Fig. 2a), more substantial influences could be seen on germination rate (*t*_50_). Deviations from favourable temperature ranges for germination increased time to reach *G*_max_ and variability in estimates for time to reach *G*_max_ in all four taxa (Fig. 3a). It is possible that, at least insofar as understanding thermal constraints, maximum germination is a less informative functional trait (Saatkamp *et al.*, 2019) than aspects of germination rate, and that, given a long enough window of opportunity, most non-dormant seeds will obtain high germination rates across a range of “sub-optimal” conditions, and it is the length of this window of opportunity that represents the selection pressure for thermal and drought tolerance in germination.

The *T*_opt_ estimates for all four species were within a range of 17–26°C, and *T*_max_ values were between 29 and 32 °C (Fig. 4a). Locations from which seeds for this study were collected are in a Mediterranean climate, characterized by hot dry summers and mild wet winters (Bell *et al.*, 1993; Fig. 1). Consequently, it has been postulated that persistence of high soil moisture availability due to frequent rainfall events from late autumn through to early spring combined with low temperatures is likely to facilitate germination and seedling establishment of most local native species at this time of year (Bell *et al*., 1993). The *T*_opt_ and *T*_max_ estimates for the four taxa clearly reflect a preference for synchronizing germination between late autumn to early spring (Fig. 4a) and are consistent with previous reports that many *Eucalyptus* species from southwest Western Australia, including short-range endemic taxa, exhibit a low thermal optimum for germination (Bell, 1994; Bell *et al*., 1995). The coincidence of germination with periods of highest rainfall among species from Mediterranean climates is widely regarded as an adaptive mechanism for summer drought avoidance when conditions are far less favourable for supporting seedling growth and establishment (Luna *et al*., 2012; Clemente *et al*., 2017), and the data that we present here indicate that it can be parameterized according to the principles of thermal biology, at least insofar as rate-related germination traits are concerned.

Exposure to water stress reduced mean final germination percentages in all species tested in this study (Fig. 2b), consistent with previous studies of *Eucalyptus* species (Pearce *et al.*, 1990), and the broader Western Australia flora (Cochrane, 2018; Turner *et al*., 2018).

### Patterns of distribution size and endemism

In terms of final germination percentage, the two range-restricted endemic taxa were more tolerant of both thermal stress, represented by higher *T*_opt_, and water stress, represented by lower *w_c_*, compared to their widespread congenerics (Fig. 2), but had narrower ranges of thermal tolerance in terms of *r*_max_ (Fig. 4a). Of the four species, the broadly distributed *E. salmonophloia* and *E. salubris* were the most drought-sensitive, with critical thresholds at −0.1 and −0.4 MPa, respectively, compared to the critical threshold for *E. caesia* subsp. *caesia* and *E. ornata* at −0.7 MPa (Fig. 3b). We suggest that these adaptations to water stress relate to the below-ground environments that characterize the species' preferred habitats: skeletal and shallow soils typical of rocky outcrops which retain water poorly, especially compared to the loamy soils that often surround these outcrops in Western Australia (Main, 1997; Mares, 1997). As well as generating extremely hot surface temperatures (Withers, 2000; Porembski and Barthlott, 2012), the water retention capacity of many habitats in outcrop environments is generally lower than the surrounding environment because the soils in these habitats are shallower compared to those of the surrounding matrix (Main, 1997; Mares, 1997). Furthermore, increased levels of evaporation due to high temperatures (especially in summer) can rapidly reduce the soil moisture availability of such microhabitats (Merritt *et al.*, 2007) because outcrops are less shaded than the neighbouring vegetation matrix (Withers, 2000). In addition, summer rainfall events in southwest Western Australia are sporadic and therefore insufficient to increase and maintain soil water potential at levels favourable for seed germination and persistence of seedlings of most taxa (Cochrane, 2018). These elements of the physical environment conspire to limit the window of opportunity for germination on rocky outcrops, a constraint that we did not impose in our experimental germinations. Limitation of germination response to a narrow tolerance range in terms of *r*_max_, combined with high drought tolerance in terms of *G*_max_, and time to reach *G*_max_ could be an adaptive strategy in range-restricted taxa such as *E. caesia* subsp. *caesia* and *E. ornata* to optimize recruitment success within a short period of opportunity in terms of high soil moisture availability following episodic rainfall events (Debat and David, 2001; Körner, 2003; Cochrane, 2018). *Eucalyptus salmonophloia* and *E. salubris* inhabit the relatively deep-soil environments surrounding granite outcrops (Yates *et al.*, 1994). Low thermal and drought tolerance in terms of final germination percentage and the relatively low *r*_max_ estimates that are consistent across a wide range of temperatures observed in *E. salmonophloia* and *E. salubris* may reflect a strategy for synchronizing seed germination with consistent rainfall during the cooler winter months under high and persistent soil moisture availability (Figs. 2 and 4a). Outside of the specific microhabitats of rocky outcrops, avoiding germination in summer is a strategy common among many species native to the deeper-soil environments surrounding granite outcrop habitats (Bell *et al*., 1993; Byrne and Hopper, 2008; Cochrane, 2017).

Our findings are in line with previous reports that the optimum temperature range for germination of widespread *Eucalyptus* species (in terms of final germination percentage) reflects the soil water regime of the habitat of each species (Bell *et al.*, 1993). Moreover, the results of our study are consistent with the findings of previous studies that seeds of range-restricted taxa that are limited by a narrow window of opportunity to germinate (in terms of soil moisture availability) exhibit high physiological plasticity for thermal and drought stress tolerance, whereas the germination response of broadly distributed congenerics living in less restrictive habitats is less plastic (Graves *et al.*, 1988; Giménez-Benavides *et al.*, 2005; Giménez-Benavides *et al.*, 2013). In this sense, the data reported in our study suggest that the seed germination traits of species from restricted distributions are consistent with general theories of stenothermic specialization in other taxa (Seebacher and Franklin, 2005).

### Limitations to interpretation

The experimental approach employed in this study can be utilized to identify optimum conditions and critical thresholds for germination in other species of threatened flora (Clemente *et al*., 2017). However, in order to get deeper insights in to the role of temperature and water stress as drivers of rarity and endemism, the above hypotheses require testing at the level of populations and individuals and the responses of a wider range of species should be compared (Mooney *et al.*, 1961; Felsenstein, 1985; Luna *et al*., 2012). Nevertheless, our findings are broadly consistent with results reported on the basis of larger numbers of species (Cochrane, 2017; Ruiz-Talonia *et al*., 2018).

While a phylogenetic perspective is critical in making comparative interpretations of this kind, it is also important to understand the trait in question. We characterized seed germination in terms of temperature at zero water stress, and water stress at optimal temperature, as have other authors faced with limited numbers of seed available from rare or range-restricted taxa (Turner *et al*., 2018). More correctly, seed germination responds to a dynamic hydro-thermal niche (Hardegree *et al.*, 2015), where the two factors interact. Characterising this interactive response may be more informative in a comparative sense, both within and between species. While eucalypts are canonically non-dormant, it is also important to assess the role of seed dormancy in determining variability in germination responses of other floral groups to thermal stress and drought stress as over 70% of native species possess seeds with some form of seed dormancy (Merritt *et al.*, 2007). Indeed, seed dormancy in most cases is also regulated by critical moisture and temperature thresholds working as another layer of environmental filters rendering seeds non dormant in response to specific soil conditions (Baskin and Baskin, 2003; Turner *et al*., 2018).

## Conclusions

We have established that in non-dormant taxa germination response to thermal stress is hump-shaped in terms of time to reach 50% germination (*t*_50_) and that at least some seed germination traits are consistent with broader theories of thermal biology. Water stress, however, caused an exponential increase in *t*_50_¸ and the theoretical bases of this remain to be clarified. The four species differed significantly in terms of thermal performance and the two range-restricted endemic taxa had narrower thermal tolerance ranges, implying adaptive stenothermy, than their widespread, eurythermic congenerics. The two-short range-endemics exhibited higher lability to temperature and drought stress compared to the two widespread species in terms of final germination percentage. The insights gained in this study could be beneficial for identifying thresholds for temperature and water stress tolerance in seeds of other flora of conservation concern.

## Supplementary Material

Supplementary_Data_coaa021
