# Supplementary material for: Hydrological and thermal responses of seeds from four co-occurring tree species from southwest Western Australia
Source: Conserv Physiol. 2020 Apr 30;8(1):coaa021. doi: 10.1093/conphys/coaa021 (PMC7192333; doi:10.1093/conphys/coaa021)
Supplement: Supplementary_Data_coaa021 [file supplementary_data_coaa021.docx]

**Supplementary Material**

Mean temperatures (±SE in parentheses) inside seed incubators for the duration of experiments

| **Experiment** | **Species** | **Treatment regime** | **No. of replicates** | **Temperature (℃)** |
| --- | --- | --- | --- | --- |
| Thermal performance |  | **(℃)** |  |  |
|  | *E. caesia* subsp. *caesia* | 5 | 8 | 5.339(0.042) |
|  |  | 10 | 8 | 10.925(0.001) |
|  |  | 15 | 8 | 14.935(0.027) |
|  |  | 20 | 8 | 18.665(0.019) |
|  |  | 25 | 8 | 25.197(0.007) |
|  |  | 30 | 8 | 30.197(0.02) |
|  |  | 35 | 8 | 34.102(0.036) |
|  | *E. ornata* | 5 | 8 | 5.302(0.042) |
|  |  | 10 | 8 | 11.011(0.044) |
|  |  | 15 | 8 | 14.788(0.021) |
|  |  | 20 | 8 | 18.817(0.019) |
|  |  | 25 | 8 | 25.054(0.01) |
|  |  | 30 | 8 | 30.131(0.023) |
|  |  | 35 | 8 | 34.209(0.033) |
|  | *E. salmonophloia* | 5 | 8 | 6.267(0.099) |
|  |  | 10 | 8 | 11.18(0.044) |
|  |  | 15 | 8 | 15.673(0.026) |
|  |  | 20 | 8 | 18.508(0.039) |
|  |  | 25 | 8 | 24.895(0.007) |
|  |  | 30 | 8 | 30.116(0.026) |
|  |  | 35 | 8 | 33.783(0.041) |
|  | *E. salubris* | 5 | 8 | 6.237(0.094) |
|  |  | 10 | 8 | 10.975(0.037) |
|  |  | 15 | 8 | 15.576(0.029) |
|  |  | 20 | 8 | 18.089(0.029) |
|  |  | 25 | 8 | 24.934(0.007) |
|  |  | 30 | 8 | 30.137(0.019) |
|  |  | 35 | 8 | 34.064(0.028) |
| Water stress tolerance at 20 ℃ |  | (MPa) |  |  |
|  | *E. caesia* subsp. *caesia* | 0 | 8 | 18.427(0.018) |
|  |  | -0.1 | 8 | 18.549(0.02) |
|  |  | -0.2 | 8 | 18.559(0.018) |
|  |  | -0.4 | 8 | 18.334(0.033) |
|  |  | -0.7 | 8 | 18.29(0.03) |
|  | *E. ornata* | 0 | 8 | 18.354(0.024) |
|  |  | -0.1 | 8 | 18.523(0.009) |
|  |  | -0.2 | 8 | 18.424(0.024) |
|  |  | -0.4 | 8 | 18.521(0.009) |
|  |  | -0.7 | 8 | 18.532(0.032) |
|  | *E. salmonophloia* | 0 | 8 | 18.424(0.014) |
|  |  | -0.1 | 8 | 18.532(0.009) |
|  |  | -0.2 | 8 | 18.426(0.009) |
|  |  | -0.4 | 8 | 18.537(0.006) |
|  |  | -0.7 | 8 | 18.243(0.028) |
|  | *E. salubris* | 0 | 8 | 18.437(0.01) |
|  |  | -0.1 | 8 | 18.386(0.028) |
|  |  | -0.2 | 8 | 18.267(0.024) |
|  |  | -0.4 | 8 | 18.236(0.014) |
|  |  | -0.7 | 8 | 18.752(0.032) |

Akaike information criterion comparison (AICc) of the unique parameter permutations of the curvilinear log-logistic model for the number of days since incubation and daily cumulative germination rate of four native western Australian *Eucalyptus* species at all temperature regimes (a) and water stress regimes (b).

|  | Model | Residual Deviance | AICc | df | Weight |  |
| --- | --- | --- | --- | --- | --- | --- |
| a |  |  |  |  |  |  |
|  | Convergent common curve | 8.894 | 37950.87 | 4 | 0 |  |
|  | Species | 7.635 | 36352.77 | 13 | 0 |  |
|  | Temperature regime | 5.630 | 33154.56 | 22 | 0 |  |
|  | Species + temperature regime | 2.608 | 25106.87 | 76 | 1 |  |
| b |  |  |  |  |  |  |
|  | Convergent common curve  Species  Water stress regime  Species + water stress regime | 9.273  7.981  2.664  6.503 | 28589.64  27422.31  25819.59  18869.09 | 4  13  16  61 | 0  0  0  1 |  |
